# Anxiety Disorders in Adults with Autism Spectrum Disorder: A Population-Based Study

**DOI:** 10.1007/s10803-019-04234-3

**Published:** 2019-10-16

**Authors:** Victoria Nimmo-Smith, Hein Heuvelman, Christina Dalman, Michael Lundberg, Selma Idring, Peter Carpenter, Cecilia Magnusson, Dheeraj Rai

**Affiliations:** 1 Centre for Academic Mental Health, Population Health Sciences, Bristol Medical School, University of Bristol, Oakfield House, Oakfield Grove, Bristol, BS8 2BN UK; 2 Avon & Wiltshire Partnership NHS Mental Health Trust, Bristol, UK; 3 Department of Public Health Sciences, Karolinska Institutet, Stockholm, Sweden; 4 Centre for Epidemiology and Community Medicine, Stockholm County Council, Stockholm, Sweden

**Keywords:** Autism spectrum disorder, Intellectual disability, Anxiety, Mental health, Epidemiology

## Abstract

Anxiety is common in children with ASD; however, the burden of specific anxiety disorders for adults with ASD is under-researched. Using the Stockholm Youth Cohort, we compared anxiety disorder diagnoses among autistic adults (n = 4049), with or without intellectual disability, and population controls (n = 217,645). We conducted additional sibling analyses. Anxiety disorders were diagnosed in 20.1% of adults with ASD compared with 8.7% of controls (RR = 2.62 [95% CI 2.47–2.79]), with greatest risk for autistic people without intellectual disability. Rates of almost all individual anxiety disorders were raised, notably obsessive–compulsive disorder and phobic anxiety disorders. Anxiety disorders were more common in full siblings and half-siblings of people with ASD. The implications of this are explored.

**Electronic supplementary material:**

The online version of this article (10.1007/s10803-019-04234-3) contains supplementary material, which is available to authorized users.

## Introduction

Autism spectrum disorders (ASD) are characterised by early-onset difficulties in social interaction, communication and restricted, repetitive patterns of interests and behaviour (Lai et al. 2014). At least 1% of the population have ASD (Brugha et al. 2016; Idring et al. 2015; Fombonne 2009). Despite a growing number of clinical and epidemiological studies, there is still limited understanding of the adult outcomes of people with ASD. Psychiatric comorbidity in ASD is associated with worse outcomes for individuals (Gillberg et al. 2016) and a greater burden for their families and society (Baxter et al. 2015; Buescher et al. 2014). Understanding the psychiatric comorbidities which affect adults with ASD is therefore essential to the planning of community services and optimising quality of life (Bakken et al. 2010; Charlot et al. 2008; Gillott and Standen 2007; La Malfa et al. 2007; Bradley and Bolton 2006).

While anxiety disorders are known to be common in children and adolescents with ASD (van Steensel et al. 2011; White et al. 2009; Vasa and Mazurek 2015) less is known about the prevalence of anxiety disorders in adult populations. Studies to date of adults provide an inconsistent account with prevalence estimates ranging between 28% and 77% and are limited by differences in methodological design, small sample size (Mazefsky et al. 2008; Tani et al. 2012), recruitment of selected samples from secondary services, or lack of a valid comparison group (Tani et al. 2012; Kanai et al. 2011; Bakken et al. 2010; Hutton et al. 2008; Buck et al. 2014; Hofvander et al. 2009; Russell et al. 2016; Lever and Geurts 2016). The existing evidence is therefore difficult to generalise and may be subject to confounding and selection bias.

The largest study of this topic to date used data from a medical insurance registration database in California and found that 29% of 1507 adults with a diagnosis of ASD also had an anxiety disorder diagnosed compared with 9% of a non-autistic reference population (adjusted odds ratio 3.69 [95% CI 3.11 to 4.36]) (Croen et al. 2015). However, other than obsessive–compulsive disorders (OCD), the study did not identify subtypes of anxiety and did not adjust for potentially important confounders such as parental mental illness or socioeconomic characteristics. A large Danish study reported over a two-fold incidence of OCD in individuals who had been diagnosed with ASD compared with those who had not (incidence rate ratio 2.18 [95% CI 1.91 to 2.48]) (Meier et al. 2015). Apart from OCD there have been no known large studies of specific anxiety disorders in adults with ASD.

It has been suggested that common mental disorders could be more prevalent in individuals with ASD without intellectual disability (ID), because they may have more insight into their problems. This pattern has been observed for depression (Rai et al. 2018) and for OCD (Meier et al. 2015) in higher functioning individuals with ASD. To date, studies of the prevalence of other anxiety disorders in adults with autism and ID have predominantly focussed on comparing individuals with intellectual disability with and without autism, with variable results regarding whether autism increases the rates of anxiety disorders, although these studies have been relatively small (Bakken et al. 2010; Bradley and Bolton 2006; Charlot et al. 2008; Gillott and Standen 2007). There is relatively more evidence on this issue in the literature on children and adolescents. For example, in their meta-analysis, van Steensel et al. found that studies of children and adolescents with ASD with a lower mean IQ had higher rates of social anxiety and generalised anxiety disorder, whilst studies with a higher mean IQ had higher prevalence of separation anxiety and OCD (lower and higher defined by the cross study mean of 87) (van Steensel et al. 2011). Only one study reported a mean IQ < 70 in which higher anxiety scores were associated with higher IQ and the presence of functional language use, however not for simple phobia, panic disorder and social phobia (Sukhodolsky et al. 2008). Further information on whether the risk of being diagnosed with anxiety disorders in the autistic population differs by the presence or absence of intellectual disability is therefore warranted.

It is possible that ASD and anxiety are associated through shared genetic causes. Genetic variants with pleiotropic effects on the ASD and anxiety phenotypes have been identified (Geschwind 2011) and family-based studies suggest aggregation of ASD in offspring to parents with anxiety disorders (Meier et al. 2015; Duvekot et al. 2016) as well as aggregation of anxiety in family members of individuals with ASD or autistic traits (Bolton et al. 1998; Jokiranta-Olkoniemi et al. 2016). However, the associations found in family-based studies may also reflect the psychological burden of having a close family member with ASD or difficulties in social interaction or communication.

In as far as ASD and anxiety are genetically linked, one could expect risk of anxiety to vary with genetic distance from the ASD proband, as it has been observed for family history (Xie et al. 2019). That is, risk of anxiety disorders would be highest in ASD cases, lower in their non-autistic full siblings (with whom they share on average 50% of their genome), lower still in their non-autistic half-siblings (with whom they share only 25% of their genome) and lowest in a non-autistic reference population (to whom they are likely unrelated). Comparing risks in this way can therefore help to explore a potential genetic correlation between autism and anxiety.

An additional advantage to the sibling design is that it allows for the direct comparison of ASD probands with non-autistic sibling controls, with whom they are likely to share many potentially confounding characteristics. Since associations within sibling pairs will not be confounded by characteristics that are shared between them (e.g. early life environment), sibling comparisons can account for unobserved confounders and may thereby provide a better estimate of causal effects when using observational data.

Using data from a large population-based cohort, this study aimed to: (1) describe the lifetime prevalence of diagnosed anxiety disorders in addition to prevalence in those aged 18 and over in people with ASD; (2) compare the risk of diagnosis of anxiety disorders in young adulthood among those with ASD (any; with ID; without ID) with that in a non-autistic reference population; (3) compare risk for diagnosis of anxiety disorders across ASD cases; their full siblings; their half-siblings; and a reference population; and (4) compare risk of anxiety disorders in adults with ASD directly with the risk of anxiety disorders among their non-autistic full siblings.

## Methods

### Study Setting and Design

**Fig. 1:**
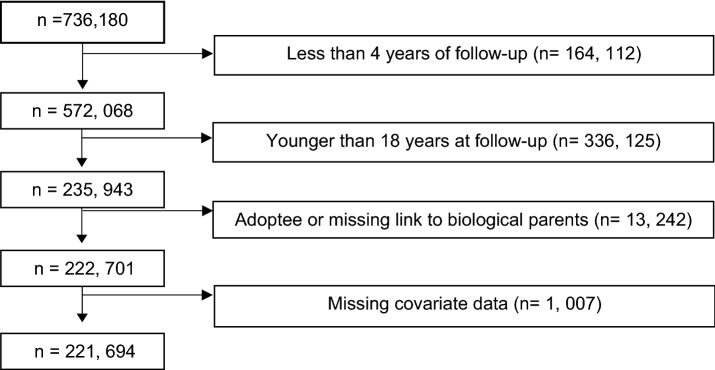
Selection procedure for the study population

The Stockholm Youth Cohort (SYC) is a register based cohort of all individuals who lived in Stockholm County, Sweden, for at least 1 year between January 1st 2001 and December 31st 2011 and were aged between 0 and 17 years at any time during that period (n = 736,180) (Idring et al. 2012, 2015). All residents in Sweden receive a unique personal identification number at birth or on immigration to Sweden, which enables the collection of prospectively recorded data via a number of national and regional health care, social and administrative registers (Idring et al. 2012). Statistics Sweden carried out the record linkages and, before the research group were given access, a serial number was used to replace the personal identity number to ensure the anonymity of cohort members. For this study, we excluded adoptees, those whose biological parents could not be identified, and those with missing covariate data. Over 96% of those with missing data were attributable to individuals or their parents having been born abroad, indicating their absence from Sweden and thus having missing information in the registers. Considering < 5% were excluded due to missing data, and there was no evidence of a difference between anxiety disorder diagnoses in those with or without missing data (p = 0.14), bias due to missing data is unlikely in this study. We included those who had been in the cohort over 4 years at the end of follow-up on December 31st 2011 for the analysis of lifetime prevalence (Supplement Figure I) and in the main analysis excluded anyone who was under the age of 18 (Fig. 1). After exclusions, our study population consisted of 221,694 aged ≥ 18 years, of whom 4049 had been diagnosed with ASD. We also identified the full and half-siblings of individuals with ASD within our study population. The study was approved by the ethical review board of Karolinska Institutet, Stockholm.

### Identification of ASD

ASD diagnoses were established using national and regional registers that encompass all known pathways for ASD diagnosis and follow-up in Stockholm County. We obtained diagnoses of ASD (ASD: ICD-9 299, ICD-10 F84, DSM-IV 299) and intellectual disability (ID: ICD-9 317-319, ICD-10 F70-79, DSM-IV 317-319), identifying individuals with ASD without ID and with ID within our study population and additionally used service use data to identify ASD and intellectual disability (Idring et al. 2012, 2015). This method has been shown to have good validity in identifying individuals with a diagnosis of ASD (Idring et al. 2012) and provides comprehensive coverage of in-patient, out-patient and primary care contacts in this population.

### Identification of Anxiety Disorders

For the purpose of this study, we included neurotic, stress related and somatoform disorders as defined in the World Health Organisation’s ICD-10 as our outcomes. We used three registers to identify members of the cohort who received a diagnosis of anxiety disorder by a clinician during the time period studied. These include the (1) National patient register, which contains the dates and discharge diagnoses of all inpatients in Sweden since 1973, and outpatient care since 2001; (2) the Stockholm adult psychiatric outpatient register which records the dates and diagnoses for any contact with specialist outpatient psychiatric services in Stockholm County since 1997; and (3) the Stockholm child and adolescent mental health register (PASTILL) which records dates and diagnoses of all child and adolescent mental health care in Stockholm County since 1999. The majority of the mental health services in Sweden are publicly funded with diagnoses contemporaneously recorded using ICD-10 codes in the relevant registers. Clinicians follow international and national guidelines when making such diagnoses, although individual variation in the diagnostic process is likely and the details of measures or tools used are not recorded in the registers. Diagnoses recorded in Swedish registers have been extensively used in psychiatric epidemiology and several validation studies on a range of psychiatric diagnoses have been carried out including obsessive compulsive disorders (Ludvigsson et al. 2011; Ruck et al. 2015; Allebeck 2009). We used the adult and child diagnostic registers to enable us to assess lifetime prevalence in addition to prevalence in those over 18 years of age. We then stratified our primary outcome variable (any anxiety disorder: ICD-10 F40-48) by diagnostic subtype to identify specific disorders as detailed in Supplement Table 1.

### Covariates

We identified variables that, in the literature, have been associated with ASD as well as anxiety. These include the individual’s age and sex, parental age at birth, highest education of either parent (≤ 9 years, 10–12, ≥ 13 years), quintile of household income, individual or parental birth country outside of Sweden, and having a record of parental mental illness (Lofors et al. 2006; de Graaf et al. 2002; Rai et al. 2012; Jokiranta et al. 2013).

### Statistical Analysis

Analyses were performed in Stata/MP version 14.2. We examined the characteristics of our study cohort by exposure status (no ASD; any ASD, further dichotomised into ASD with and without ID) in relation to the outcomes. We then used modified Poisson regression to estimate the relative risk (RR) of being diagnosed with an anxiety disorder after the age of 18 among those with a diagnosis of ASD (any; with ID; without ID) compared with a non-autistic reference population, using robust standard errors to account for clustering of outcomes within families. We compared the relative risk estimates for anxiety disorder diagnosis for adults with ASD with and without comorbid ID in supplementary analysis. In supplementary analysis we also calculated the prevalence of anxiety disorders for those with ASD (any; with ID; without ID) for the full population of those aged 4–27 years of age. To explore a potential genetic gradient, we also estimated the relative risk of anxiety disorders among the non-autistic full- and half-siblings of ASD cases compared with a non-autistic reference population using the same methods (but clustering on maternal rather than family identification number for half-siblings), and compared these relative risk estimates in supplementary analysis. We adjusted our estimates for (1) sex and age at the end of follow-up, and additionally for (2) parental age, parental educational attainment, family disposable income quintile, individual or parental foreign birth, and maternal and paternal psychiatric history. To assess the extent to which associations between ASD and anxiety were due to unobserved shared familial confounders, we directly compared adult individuals with ASD with their discordant full sibling controls using conditional logistic regression models with adjustment for non-shared characteristics between sibling pairs (age, birth order, sex, maternal and paternal age). Lastly, as anxiety disorders are known to be a common problem in children with ASD (van Steensel et al. 2011), we wanted to assess whether anxiety disorders in adults were a continuation of these, or a separate clinical issue which requires its own consideration. For this reason, we identified the relative risk of a new diagnosis of an anxiety disorder in adulthood in people with no previous history of an anxiety disorder as this could provide clinically relevant information for clinicians working with adults. As further clinically useful information, in a supplementary analysis, we calculated the average age of onset of these anxiety disorders and the proportion of the sample diagnosed with anxiety before and/or after the age of 18.

## Results

Our cohort contained 221,694 individuals aged 18 to 27 years of whom 4049 had been diagnosed with ASD. We provide study characteristics and descriptive statistics by ASD case status, including the lifetime prevalence of a diagnosis of specific types of anxiety disorders, in Table 1. Just over a fifth (20.13%) of adults with ASD had also been diagnosed with an anxiety disorder compared with 8.72% of an adult non-autistic reference population (adjusted RR 2.62 [95% CI 2.47–2.79]), and prevalence of anxiety disorder was highest in the absence of comorbid ID (23.11%) (Tables 1 and 2).

**Table 1:** Characteristics of the total study population (age 18–27) by ASD diagnosis and ASD with and without intellectual disability

	Presence of ASD diagnosis			
	None (n = 217,645)	Any (n = 4049)	ASD without ID (n = 2908)	ASD with ID (n = 1141)
Characteristic				
Age range 18–27. Mean (sd)	22.66 (2.86)	21.97 (2.71)	21.95 (2.68)	22.04 (2.80)
Male sex (%)	50.89	65.94	64.44	69.76
Mother’s age at birth. Mean (sd)	28.64 (5.27)	29.16 (5.64)	29.03 (5.59)	29.47 (5.75)
Father’s age at birth. Mean (sd)	31.71 (6.32)	32.08 (6.70)	31.83 (6.64)	32.72 (6.80)
Parental education < 9 years (%)	9.46	8.22	7.26	10.69
Family income in lowest quintile (%)	22.45	19.51	17.64	24.28
Individual or their parents born outside Sweden (%)	35.05	31.39	28.37	39.09
Maternal psychiatric history (%)	35.03	49.49	51.86	43.47
Paternal psychiatric history (%)	22.90	31.51	32.26	29.62

	Prevalence % (n)			
All anxiety disorders	8.72 (18,980)	20.13 (815)	23.11 (672)	12.53 (143)
All phobic anxiety disorders	0.90 (1968)	3.75 (152)	4.71 (137)	1.31 (15)
Social phobia	0.63 (1369)	3.04 (123)	3.95 (115)	0.70 (8)
Agoraphobia	0.23 (508)	0.74 (30)	0.93 (27)	0.26 (3)
Specific phobia	0.10 (213)	0.20 (8)	0.24 (7)	0.09 (1)
Other phobia	0.05 (115)	0.27 (11)	0.14 (4)	0.61 (7)
Panic disorder	1.69 (3688)	2.91 (118)	3.44 (100)	1.58 (18)
GAD	0.60 (1304)	1.65 (67)	2.06 (60)	0.61 (7)
OCD	0.47 (1017)	3.43 (139)	3.85 (112)	2.37 (27)
Acute stress reaction	0.70 (1524)	1.98 (80)	2.30 (67)	1.14 (13)
PTSD	0.30 (643)	0.74 (30)	0.93 (27)	0.26 (3)
Adjustment disorders	0.42 (919)	1.28 (52)	1.41 (41)	0.96 (11)
Other stress-related disorders	2.23 (4861)	2.03 (82)	2.34 (68)	1.23 (14)
Dissociative disorder	0.04 (88)	0.32 (13)	0.21 (6)	0.61 (7)
Somatoform disorders	0.25 (546)	0.62 (25)	0.72 (21)	0.35 (4)
Mixed anxiety/depression	1.31 (2860)	4.74 (192)	5.85 (170)	1.93 (22)
Other neurotic or anxiety disorder	3.68 (7999)	8.13 (329)	9.28 (270)	5.17 (59)

*GAD* generalised anxiety disorder, *OCD* obsessive–compulsive disorder, *PTSD* post-traumatic stress disorder

**Table 2:** Relative risk of a diagnosis of anxiety disorder between ages 18 and 27 years in individuals with ASD, and ASD with or without ID compared to the general population

	All ASD (n = 4049)		ASD without ID (n = 2908)		ASD with ID (n = 1141)	
	Adjusted RR Model 1^a^	Adjusted RR Model 2^b^	Adjusted RR Model 1^a^	Adjusted RR Model 2^b^	Adjusted RR Model 1^a^	Adjusted RR Model 2^b^
All anxiety disorders	2.95 (2.78–3.14)	2.62 (2.47–2.79)	3.38 (3.16–3.60)	2.96 (2.77–3.16)	1.87 (1.60–2.17)	1.71 (1.47–1.99)
All phobic anxiety disorders	5.19 (4.41–6.10)	4.54 (3.85–5.35)	6.48 (5.48–7.67)	5.57 (4.69–6.61)	1.84 (1.11–3.04)	1.68 (1.01–2.79)
Social phobia	5.86 (4.88–7.03)	5.07 (4.21–6.10)	7.60 (6.30–9.16)	6.46 (5.34–7.82)	1.35 (0.68–2.70)	1.23 (0.61–2.45)
Agoraphobia	4.10 (2.84–5.91)	3.53 (2.44–5.11)	5.10 (3.47–7.49)	4.31 (2.92–6.36)	1.49 (0.48–4.62)	1.35 (0.44–4.21)
Specific phobia	3.00 (1.49–6.07)	2.77 (1.37–5.59)	3.61 (1.70–7.64)	3.28 (1.55–6.93)	1.40 (0.20–9.93)	1.34 (0.19–9.57)
Other phobia	6.48 (3.50–12.01)	5.99 (3.20–11.19)	3.29 (1.21–8.97)	2.98 (1.10–8.10)	14.92 (7.03–31.66)	13.93 (6.51-29.78)
Panic disorder	2.20 (1.84–2.63)	1.97 (1.65–2.37)	2.58 (2.13–3.14)	2.29 (1.89–2.80)	1.20 (0.76–1.90)	1.11 (0.70–1.76)
GAD	3.68 (2.88–4.69)	3.17 (2.48–4.05)	4.55 (3.52–5.87)	3.84 (2.97–4.97)	1.39 (0.66–2.90)	1.27 (0.61–2.65)
OCD	9.11 (7.64–10.85)	8.07 (6.74–9.65)	10.21 (8.43–12.38)	8.88 (7.28–10.82)	6.24 (4.28–9.10)	5.78 (3.96–8.44)
Acute stress reaction	3.71 (2.97–4.64)	3.24 (2.58–4.07)	4.30 (3.38–5.47)	3.72 (2.91–4.75)	2.17 (1.26–3.73)	1.93 (1.12-3.31)
PTSD	3.47 (2.41–5.00)	2.98 (2.06–4.32)	4.27 (2.91–6.26)	3.66 (2.48–5.39)	1.28 (0.41–3.96)	1.10 (0.36–3.44)
Adjustment disorders	3.98 (3.02–5.25)	3.49 (2.65–4.60)	4.37 (3.20–5.95)	3.79 (2.78–5.17)	3.01 (1.66–5.44)	2.73 (1.51–4.93)
Other stress-related disorders	1.26 (1.02–1.57)	1.11 (0.90–1.38)	1.45 (1.15–1.84)	1.26 (1.00–1.60)	0.78 (0.46–1.31)	0.70 (0.42–1.18)
Dissociative disorder	11.02 (6.13–19.81)	10.12 (5.59–18.32)	6.94 (3.04–15.82)	6.45 (2.76–15.08)	22.04 (10.11–48.02)	20.42 (9.46–44.11)
Somatoform disorders	3.11 (2.08–4.65)	2.93 (1.95–4.41)	3.64 (2.35–5.64)	3.42 (2.20–5.33)	1.75 (0.65–4.68)	1.64 (0.62–4.40)
Mixed anxiety/depression	4.65 (4.03–5.36)	3.98 (3.45–4.60)	5.66 (4.88–6.57)	4.75 (4.08–5.53)	1.95 (1.29–2.96)	1.76 (1.16–2.67)
Other neurotic or anxiety disorder	2.88 (2.59–3.19)	2.53 (2.28–2.81)	3.28 (2.93–3.67)	2.84 (2.54–3.19)	1.85 (1.44–2.36)	1.68 (1.32–2.15)

*GAD* generalised anxiety disorder, *OCD* obsessive–compulsive disorder, *PTSD* post-traumatic stress disorder, *RR* relative risk, *CI* confidence interval

^a^Modified Poisson regression with cluster robust standard errors. Model 1 adjusted for age and sex

^b^Model 2 adjusted for age, sex, maternal and paternal age, parental educational attainment, family disposable income quintile, individual or parental migration, and maternal and paternal psychiatric history

The most common diagnostic labels were non-specific, with 5.91% of the reference population and 10.16% of adults with ASD having a diagnosis of “other stress related disorders” or a non-specific anxiety disorder diagnosis, which fell under our category label of “other neurotic or anxiety disorder” (Table 1). Except for “other stress-related disorders”, people with ASD had higher relative risks for receiving a diagnosis of all types of anxiety disorder compared to a non-autistic reference population (Table 2). Amongst the common anxiety disorder diagnoses, prevalence of OCD diagnoses was notably raised in people with ASD (3.43%) compared with the general population (0.47%) (adjusted RR 8.07 [95% CI 6.74–9.65]) and prevalence of phobic anxiety disorders was also markedly higher. This increase can be partly accounted for by an increased prevalence of social phobia for adults with ASD; however, there were also higher rates of all other phobic anxiety disorders. The relative risk of developing a dissociative disorder for an adult with ASD was also high; however, the numbers of individuals with this disorder were low, leading to very wide confidence intervals.

There was a clear difference between the rates of anxiety disorders diagnosed in adults with ASD associated with the presence or absence of intellectual disability (Table 2). The relative risk for an anxiety disorder diagnosis for adults with ASD without ID was almost three times higher than the general population (adjusted RR 2.96 [95% CI 2.77–3.16]), higher than the same estimate for adults with ASD with ID (adjusted RR 1.71 [95% CI 1.47–1.99]) (Table 2), with evidence of a statistical difference in these estimates (p < 0.001, Supplement Table 3). Adults with ASD without ID had higher adjusted risks of panic disorder, generalised anxiety disorder, PTSD, somatoform disorders and mixed anxiety and depression, trends which were not present for those with ASD and ID. Rates of diagnoses of specific phobias, such as social phobia and agoraphobia were also higher for adults with ASD without ID, whereas adults with ASD who had ID were more likely to receive the non-specific diagnosis of ‘other phobia’. Mixed anxiety and depression, adjustment disorders and acute stress reactions were diagnoses which were more common in adults with ASD both with and without ID compared to the non-autistic reference population.

Comparing the ASD cases with their non-autistic full- and half-siblings, risk of anxiety disorder was highest amongst the ASD cases than their full- and half-siblings, and risks were raised in siblings compared with the general population. However, we did not observe a consistent gradient in risk with increased genetic distance (Table 3) although there was some evidence of such a difference in ASD without ID (p = 0.021, Supplement Table 4). Risk for anxiety among cases was highest in the absence of comorbid ID, although risk of anxiety among siblings did not appear to vary with being the family member of person with ASD with or without ID.

**Table 3:** Relative risk (95% CI) for a diagnosis of an anxiety disorder among those diagnosed with ASD, their siblings and half-siblings, compared with population controls

	Cases versus population controls		Siblings of cases versus population controls		Half-siblings of cases versus population controls	
	RR (95% CI) Model 1^a^	RR (95% CI) Model 2^b^	RR (95% CI) Model 1^a^	RR (95% CI) Model 2^b^	RR (95% CI) Model 1^a^	RR (95% CI) Model 2^b^
All ASD	2.95 (2.78–3.14)	2.62 (2.47–2.79)	1.50 (1.38–1.64)	1.37 (1.26–1.49)	1.48 (1.31–1.68)	1.22 (1.07–1.38)
ASD without ID	3.38 (3.16–3.60)	2.96 (2.77-3.16)	1.59 (1.44–1.76)	1.41 (1.28–1.57)	1.41 (1.22–1.62)	1.15 (1.00–1.33)
ASD with ID	1.87 (1.60–2.17)	1.71 (1.47–1.99)	1.32 (1.11–1.56)	1.28 (1.08–1.51)	1.77 (1.37–2.28)	1.48 (1.14–1.92)

*RR* relative risk, *CI* confidence interval

^a^Modified Poisson regression with cluster robust standard errors. Model 1 adjusted for age and sex

^b^Model 2 adjusted for age, sex, maternal and paternal age, parental educational attainment, family disposable income quintile, foreign birth of child or parents, and maternal and paternal psychiatric history

We then directly compared risk of anxiety in adults with ASD with risk in their discordant non-autistic full siblings using a conditional logistic model. Cases with ASD had a higher risk of anxiety disorders compared with their discordant non-autistic full siblings (adjusted OR 2.48 [95% CI 2.06–3.01]) suggesting that associations were robust against confounding from factors shared between siblings. Associations were stronger among full siblings discordant for ASD without comorbid ID (adjusted OR 3.10 [95% CI 2.50–3.85]). Amongst siblings discordant for ASD with comorbid ID there did not appear to be an association between ASD diagnosis and anxiety disorder (adjusted OR 1.04 [95% CI 0.71–1.54]).

The mean age of diagnosis for all anxiety disorders was just under the age of 15 for people with ASD and just under the age of 17 for people without ASD (Supplement Table 5). However, our research found that even when there had been no history of a childhood anxiety disorder (66% of all ASD cases), adults with ASD continued to be at higher risk of being diagnosed with an anxiety disorder compared to adults without ASD (adjusted RR 2.71 [95% CI 2.52–2.91]) and this risk was highest for people with ASD without ID (adjusted RR 3.13 [95% CI 2.90–3.38]) (Supplement Tables 6 and 7).

## Discussion

In this large population based study in Sweden, we found that people with ASD were over two and a half times more likely to have a diagnosis of an anxiety disorder than a reference population without ASD, and risk appeared highest for people with ASD without intellectual disability.

There are only a few epidemiological studies with which we can directly compare our results. Our findings of higher rates of anxiety disorders in general and OCD specifically are consistent with evidence in adults found in previous studies (Croen et al. 2015; Meier et al. 2015). This is the first large, population-based study which has assessed the rates of PTSD in adults with ASD. This is consistent with a large study of autistic traits amongst nursing staff which found that PTSD rates were highest amongst those in the highest quintile for autistic traits (10.7%) versus those in the lowest quintile for autistic traits (4.5%) (Roberts et al. 2015). Previous research has additionally suggested that traumatic childhood events are associated with developmental disorders, including ASD, but that low reported rates of PTSD in youth with ASD may be due to diagnosis being complicated by individuals with ASD manifesting symptoms of traumatic stress in a distinct manner from individuals without ASD (Kerns et al. 2015).

It is of note that the most commonly used diagnostic labels for all individuals were those identifying a non-specified anxiety or neurotic disorder, which we termed “other neurotic or anxiety disorder”, and there were a significantly greater number of individuals with ASD being given the diagnosis of “other phobias” than the general population. This may be due to specific disorders or phenomenology being harder to distinguish in individuals with ASD, or due to multiple phobic anxiety disorders being present which may complicate the diagnostic picture. To our knowledge this is the first study to measure somatoform disorder and dissociative disorder in individuals with ASD, and our study found significantly higher rates of these conditions.

Higher rates of anxiety disorders in people with ASD may occur for a number of reasons (Kerns and Kendall 2012). For example, people with ASD may be more likely to experience peer rejection and prevention or punishment of their desired behaviours (for example restricted, repetitive interests). Additionally, social difficulties such as repeated experiences of misinterpreting social situations or communication leading to misunderstandings may produce anxiety, particularly in social situations. In typically developing adolescents and those with ASD, social difficulties have been associated with increased anxiety (Bellini 2006) and in particular an individual’s perception of their social skills difficulties is predictive of social anxiety (Bellini 2004). The impact of social stressors may be increased by a biological vulnerability to anxiety. For example, limbic system dysfunction and behavioural inhibition are associated with both ASD and anxiety disorders. Lower arousal thresholds in the amygdala associated with behavioural inhibition may in turn result in people with ASD avoiding and being conditioned by negative experiences (Bellini 2006). Sensory over-responsivity has also been suggested as a possible cause of anxiety disorder in ASD (Mazurek et al. 2013), causing problematic fears to develop as a result of increased sensitivity to certain stimuli. In OCD, whilst obsessional thoughts are common in the general population, it has been suggested that the cognitive deficits associated with ASD may influence the manner in which these thoughts are appraised, resulting in more anxiety and the development of OCD (Russell et al. 2005).

To our knowledge this is the first study of the difference in prevalence rates of anxiety disorders for adults with ASD with and without ID. The finding of higher rates of anxiety disorders amongst adults with ASD without ID compared with adults with ASD and ID is consistent with some of the results from studies of children with ASD and anxiety disorder (Weisbrot et al. 2005; Sukhodolsky et al. 2008; Mayes et al. 2011) and with a recent study of depression in ASD (Rai et al. 2018), and may be due to a number of factors. Increased rates of anxiety disorders may be present in individuals without ID due to increased cognitive awareness of their impairments (Bauminger et al. 2003). It may also be that the rates of anxiety disorder in people with ASD and ID are underestimated due to carers or clinicians attributing features of anxiety as a symptom of an individual’s ID, also known as diagnostic overshadowing. We found higher rates of “other phobias” in individuals with ASD and ID, consistent with studies in youth, where ID was associated with more “atypical” versus “traditional” anxiety features (Kerns et al. 2014). Additionally, given that many anxiety symptoms are associated with increased verbal ability, the absence of an ID and associated increase in verbal skills may play a key role in the ability to communicate anxiety symptoms. This is consistent with studies in children and adolescents where the presence of functional language use has been associated with an increase in anxiety symptoms in children with ASD (Sukhodolsky et al. 2008), and has been shown to have an opposite pattern to children without ASD, where language deficits were associated with increased anxiety symptoms, suggesting a unique relationship between ASD, language and anxiety (Davis et al. 2011).

Assessing the risk of anxiety disorders in full- and half-siblings of individuals can be a useful tool to help find gradients in risk that may be influenced by genetic loading. Our results did not identify any evidence of risk gradients between full- and half-siblings of individuals with ASD; however, there was an increased risk of an anxiety disorder diagnosis for siblings and half-siblings compared with the general population. Studies of full-siblings of children and young adults with ASD have found a comparable increased risk of anxiety disorders (Jokiranta-Olkoniemi et al. 2016). However, we were not able to control for the situations such as parental separation which may contribute to higher rates of anxiety disorders in all half-siblings, possibly masking a gradient. While the increased risk of anxiety in siblings may still suggest the role of common genetic vulnerability, siblings of children with ASD may also be prone to psychiatric disorders through other mechanisms. For example, they may receive less parental attention because of their autistic sibling having more needs or may experience other stressors in the household. The sibling control analysis allowed us to directly compare the risk of anxiety disorder in individuals with ASD versus their discordant siblings. This enabled additional control of shared-environmental and shared-genetic confounding factors. The notably higher rates in adults with ASD compared with their discordant siblings gives additional evidence for the prospect of genetic factors specifically related to ASD, or other ASD-specific environmental factors that increase vulnerability to anxiety disorders, which could be possible targets for future interventions.

This study highlights that anxiety disorders are a significant problem for adults with autism and there is therefore a need for effective evidence-based treatments. Cognitive-behavioural therapy (CBT) is the most researched therapy for anxiety disorders in individuals with ASD; however, whilst there is a growing volume of research supporting the use of CBT for anxiety in children and adolescents with ASD, less is known about the use of CBT in adults with ASD (Rosen et al. 2018). Concerning pharmacological treatments, current evidence suggests that selective serotonin re-uptake inhibitors (SSRIs) produce benefit in anxiety disorders in ASD, including to obsessional thoughts (Buchsbaum et al. 2001; McDougle et al. 1996) and compulsions (Hollander et al. 2012; McDougle et al. 1996). However, studies have been small and the overall evidence for pharmacological treatments is also limited (Howes et al. 2018).

The results of this study should be interpreted in the light of the following limitations. Firstly, as this is a register based study, the possibility of exposure and outcome misclassification cannot be ruled out. There may be undiagnosed people with ASD in the population which could lead to some exposure misclassification. If this were differential in relation to the outcome, for example, if those with greater anxiety symptoms were more likely to be diagnosed with ASD more easily, then our observed associations may be over-estimates of the true association. If the reverse were true or the misclassification was not differential, then the results would likely be biased towards the null. Likewise, it is likely there are individuals with anxiety disorders in our cohort who have not been recorded as such. Due to the use of registers we were also not able to verify the anxiety disorder diagnoses. This may be an important limitation as there are phenomenological differences in presentation of anxiety disorders in individuals with ASD and/or ID and there may be potential variability between clinicians and mental health care centres in their recognition. The possibility of such measurement error may be compounded by the lack of standardised tools available for the measurement of anxiety disorders within the ASD population. Such misclassification may have been somewhat minimised by our use of anxiety disorder diagnoses from secondary care and inpatient records which are made by specialists, although it should be noted that there are still no clear consensus guidelines for measuring anxiety in ASD. Although the majority of diagnoses of specific anxiety disorders such as OCD would be made in secondary care, primary care doctors are involved in diagnosing anxiety disorders, particularly generalised anxiety disorder and mixed anxiety and depression; this means that the rates of these conditions within our study may be underestimated. Therefore, the possibility of outcome misclassification cannot be ruled out. Such limitations would be common to any record-linkage study and cohort studies with detailed clinical information would need to be carried out to assess this further. We were unable to measure early life trauma, which is a limitation as it may be associated with ASD (Kerns et al. 2015) and with anxiety disorders, particularly the higher rates of PTSD. Despite these limitations, this study has several strengths. The Swedish registers are of high quality, enabling a large sample size with the ability to adjust for a range of confounders and examine siblings of individuals with ASD. The prospective data collection minimises the possibility of recall bias.

## Conclusion

Anxiety disorders are a notable problem for people with ASD. It is important for adult mental health services to be skilled at working with this population where co-occurring anxiety is so prevalent. More research is needed to determine the causes of increased anxiety for people with ASD. Our findings suggest a potential for environmental pathways; however, delineating these further may help the development of preventative interventions or targeted treatment. Our study has identified increased rates of specific anxiety disorders for adults with ASD which has been explored in children and adolescents but not studied previously in adults and these associations need to be replicated in future studies. Future research is also needed to better understand the phenomenology of anxiety disorders in this population and to better the means of measuring and treating anxiety in this population. This may be an aim worth pursuing because effective management of anxiety in this population could lead to major gains in quality of life and functioning (Kerns and Kendall 2012).

## Electronic supplementary material

Below is the link to the electronic supplementary material.

Supplementary material 1 (DOCX 46 kb)
