# Supplementary material for: Anxiety Disorders in Adults with Autism Spectrum Disorder: A Population-Based Study
Source: J Autism Dev Disord. 2019 Oct 16;50(1):308–18. doi: 10.1007/s10803-019-04234-3 (PMC6946757; doi:10.1007/s10803-019-04234-3)
Supplement: Supplementary file 1 — Supplementary material 1 (DOCX 46 kb) [file 10803_2019_4234_MOESM1_ESM.docx]

**Supplementary Material**

**Supplementary Figure I. Selection procedure for the study population of 4-27 year olds**

n =736,180

n = 572, 068

Less than 4 years in the cohort (n=164, 112 )

Adoptee or missing link to biological parents (n= 24, 837)

n = 547, 231

Missing covariate data (n= 1, 671)

n = 545, 560

**Supplementary Table 1: Primary outcome variable stratified by diagnostic subtype**

|  | **ICD 10 code** |
| --- | --- |
| All anxiety disorders | F40-48 |
| All phobic anxiety disorders |  |
| Agoraphobia | F40.0 |
| Social phobia | F40.1 |
| Specific phobia | F40.2 |
| Other phobia | F40.8 |
| Panic disorder | F41.0 |
| Generalised anxiety disorder | F41.1 |
| Mixed Anxiety and depression | F41.2 |
| Obsessive-compulsive disorder | F42 |
| Acute stress reaction | F43.0 |
| Post-traumatic stress disorder | F43.1 |
| Adjustment disorders | F43.2 |
| Other stress-related disorder | F43.8 |
| Dissociative Disorder | F44 |
| Somatoform disorders | F45 |
| Other neurotic or anxiety disorder | F41.8-F41.9 and F48 |

**Supplementary Table 2: Percentage of the population aged 4-27 with a diagnosis of anxiety disorder**

|  | **Presence of ASD diagnosis** | | | |
| --- | --- | --- | --- | --- |
|  | **No ASD**  (n=534,430) | **Any**  (n=11,130) | **ASD without ID**  (n=8,428) | **ASD with ID**  (n=2,702) |
|  | % (n) | % (n) | % (n) | % (n) |
| **All anxiety disorders** | 7.10 (37,970) | 21.25 (2,365) | 23.49 (1,980) | 14.25 (385) |
| All phobic anxiety disorders | 0.61 (3,274) | 2.98 (332) | 3.10 (261) | 2.63 (71) |
| Social Phobia | 0.34 (1, 803) | 1.61 (179) | 1.98 (167) | 0.44 (12) |
| Agoraphobia | 0.11 (609) | 0.36 (40) | 0.38 (32) | 0.30 (8) |
| Specific phobia | 0.13 (683) | 0.49 (55) | 0.61 (51) | 0.15 (4) |
| Other Phobia | 0.08 (435) | 0.71 (79) | 0.33 (28) | 1.89 (51) |
| Panic Disorder | 0.84 (4,490) | 1.38 (154) | 1.52 (128) | 0.96 (26) |
| GAD ^1^ | 0.34 (1,842) | 1.03 (115) | 1.22 (103) | 0.44 (12) |
| OCD ^2^ | 0.53 (2,846) | 4.28 (476) | 4.64 (391) | 3.15 (85) |
| Acute stress reaction | 0.55 (2,949) | 1.48 (165) | 1.66 (140) | 0.93 (25) |
| PTSD ^3^ | 0.47 (2,491) | 1.45 (161) | 1.66 (140) | 0.78 (21) |
| Adjustment disorders | 0.33 (1,763) | 1.13 (126) | 1.29 (109) | 0.63 (17) |
| Other stress-related disorders | 1.35 (7,241) | 1.77 (197) | 1.83 (154) | 1.59 (43) |
| Dissociative Disorder | 0.06 (306) | 0.50 (56) | 0.50 (42) | 0.52 (14) |
| Somatoform disorders | 0.39 (2,093) | 0.93 (104) | 1.04 (88) | 0.59 (16) |
| Mixed anxiety/ depression | 0.62 (3,316) | 2.08 (232) | 2.42 (204) | 1.04 (28) |
| Other neurotic or anxiety disorder | 3.49 (18,649) | 11.05 (1,230) | 12.87 (1, 085) | 5.37 (145) |

^Notes: (1) Generalised anxiety disorder. (2) Obsessive-compulsive disorder. (3) Post-traumatic stress disorder.^

**Supplementary Table 3: Comparison of relative risk estimates for ASD with and without comorbid ID in adults (aged 18-27 years)**

|  | ASD without ID  (n= 2,908) | ASD with ID  (n= 1,141) |  |
| --- | --- | --- | --- |
|  | **Adjusted RR**  **(95% CI) ^4^** | **Adjusted RR**  **(95% CI) ^4^** | **P-value ^5^** |
| All anxiety disorders | 2.96 (2.77 - 3.16) | 1.71 (1.47 - 1.99) | <0.001 |
| All phobic anxiety disorders | 5.57 (4.69- 6.61) | 1.68 (1.01- 2.79) | <0.001 |
| Social Phobia | 6.46 (5.34- 7.82) | 1.23 (0.61- 2.45) | <0.001 |
| Agoraphobia | 4.31 (2.92- 6.36) | 1.35 (0.44- 4.21) | 0.057 |
| Specific phobia | 3.28 (1.55- 6.93) | 1.34 (0.19- 9.57) | 0.401 |
| Other Phobia | 2.98 (1.10- 8.10) | 13.93 (6.51-29.78) | 0.013 |
| Panic Disorder | 2.29 (1.89- 2.80) | 1.11 (0.70- 1.76) | 0.004 |
| GAD ^1^ | 3.84 (2.97- 4.97) | 1.27 (0.61- 2.65) | 0.005 |
| OCD ^2^ | 8.88 (7.28- 10.82) | 5.78 (3.96- 8.44) | 0.044 |
| Acute stress reaction | 3.72 (2.91- 4.75) | 1.93 (1.12-3.31) | 0.029 |
| PTSD ^3^ | 3.66 (2.48- 5.39) | 1.10 (0.36- 3.44) | 0.049 |
| Adjustment disorders | 3.79 (2.78- 5.17) | 2.73 (1.51- 4.93) | 0.331 |
| Other stress-related disorders | 1.26 (1.00- 1.60) | 0.70 (0.42- 1.18) | 0.044 |
| Dissociative Disorder | 6.45 (2.76- 15.08) | 20.42 (9.46- 44.11) | 0.040 |
| Somatoform disorders | 3.42 (2.20- 5.33) | 1.64 (0.62- 4.40) | 0.179 |
| Mixed anxiety/depression | 4.75 (4.08- 5.53) | 1.76 (1.16- 2.67) | <0.001 |
| Other neurotic or anxiety disorder | 2.84 (2.54- 3.19) | 1.68 (1.32- 2.15) | <0.001 |

^Notes: (1) Generalised anxiety disorder. (2) Obsessive-compulsive disorder. (3) Post-traumatic stress disorder. (4) RR = relative risk of outcome compared with a non-autistic reference population. CI = confidence interval. Associations were estimated using modified Poisson regression with cluster robust standard errors, with statistical adjustment for age, sex, maternal and paternal age, parental educational attainment, family disposable income quintile, individual or parental migration, and maternal and paternal psychiatric history. (5) Tests comparing relative risk estimates were performed using the^ *^suest^* ^post-estimation command^

**Supplementary Table 4: Comparison of relative risk of anxiety for ASD cases, their full siblings, and half-siblings**

|  | Relative risk of anxiety for: | | |  | Comparison of relative risk estimates for: | | |
| --- | --- | --- | --- | --- | --- | --- | --- |
|  | ASD Cases | Full siblings of cases | Half-siblings of cases |  | ASD cases vs full siblings | ASD cases vs half-siblings | Siblings vs half-siblings |
|  | RR (95% CI) ^1^ | RR (95% CI) ^1^ | RR (95% CI) ^1^ |  | P-value ^2^ | P-value ^2^ | P-value ^2^ |
| All ASD | 2.62 (2.47- 2.79) | 1.37 (1.26- 1.49) | 1.22 (1.07- 1.38) |  | <0.001 | <0.001 | 0.124 |
| ASD without ID | 2.96 (2.77-3.16) | 1.41 (1.28- 1.57) | 1.15 (1.00- 1.33) |  | <0.001 | <0.001 | 0.021 |
| ASD with ID | 1.71 (1.47- 1.99) | 1.28 (1.08- 1.51) | 1.48 (1.14- 1.92) |  | 0.011 | 0.342 | 0.352 |

^Notes: (1) RR = relative risk of outcome compared with a non-autistic reference population. CI = confidence interval. Associations were estimated using modified Poisson regression with cluster robust standard errors, with statistical adjustment for age, sex, maternal and paternal age, parental educational attainment, family disposable income quintile, individual or parental migration, and maternal and paternal psychiatric history. (2) Tests comparing relative risk estimates were performed using the^ *^suest^* ^post-estimation command.^

**Supplementary Table 5: Mean age of first diagnosis of anxiety disorder**

|  | **Presence of ASD diagnosis** | | | |
| --- | --- | --- | --- | --- |
|  | **None** | **Any** | **ASD without ID** | **ASD with ID** |
|  | (n=534,430) | (n=11,130) | (n=8,428) | (n=2,702) |
| **All anxiety disorders** | 16.80 (16.75-16.86) | 14.65 (14.45- 14.86) | 14.61 (14.39- 14.83) | 14.88 (14.30- 15.46) |
| All phobic anxiety disorders | 17.98 (17.80- 18.17) | 16.19 (15.60- 16.77) | 17.13 (16.52- 17.74) | 12.72 (11.41- 14.04) |
| Social Phobia | 20.14 (20.00- 20.31) | 19.16 (18.69- 19.64) | 19.16 (18.66- 19.66) | 19.14 (17.37- 20.92) |
| Agoraphobia | 20.64 (20.38- 20.90) | 19.38 (17.66- 21.11) | 20.72 (19.26- 22.18) | 14.04 (8.24- 19.84) |
| Specific phobia | 14.70 (14.28- 15.12) | 12.45 (11.25- 13.65) | 12.44 (11.19- 13.69) | 12.59 (5.02- 20.16) |
| Other Phobia | 12.42 (11.78- 13.07) | 11.92 (10.72- 13.12) | 12.25 (10.21- 14.30) | 11.74 (10.21- 13.26) |
| Panic Disorder | 20.59 (20.50- 20.69) | 19.80 (19.23- 20.36) | 19.87 (19.26- 20.47) | 19.44 (17.83- 21.05) |
| GAD ^1^ | 19.48 (19.26- 19.71) | 17.89 (16.85- 18.93) | 17.87 (16.77- 18.96) | 18.12 (14.27- 21.96) |
| OCD ^2^ | 16.07 (15.89- 16.25) | 15.26 (14.83- 15.69) | 15.09 (14.62- 15.57) | 16.03 (15.02- 17.03) |
| Acute stress reaction | 17.26 (17.04- 17.48) | 17.23 (16.32- 18.13) | 17.46 (16.55-18.36) | 15.94 (12.65-19.23) |
| PTSD ^3^ | 14.42 (14.19- 14.65) | 12.40 (11.41- 13.40) | 12.78 (11.75- 13.80) | 9.92 (6.44- 13.39) |
| Adjustment disorders | 17.39 (17.15- 17.63) | 16.60 (15.74- 17.45) | 16.19 (15.26- 17.13) | 19.19 (17.41- 20.96) |
| Other stress-related disorders | 19.37 (19.26- 19.49) | 16.95 (16.24- 17.65) | 17.33 (16.56- 18.10) | 15.56 (13.87- 17.26) |
| Dissociative Disorder | 15.98 (15.45- 16.52) | 15.07 (13.94- 16.20) | 15.09 (13.97- 16.20) | 15.02 (11.61- 18.43) |
| Somatoform disorders | 14.51 (14.28- 14.75) | 14.89 (13.92- 15.86) | 14.96 (13.90- 16.02) | 14.48 (11.81- 17.16) |
| Mixed anxiety/ depression | 20.87 (20.77-20.97) | 20.25 (19.87- 20.62) | 20.25 (19.86- 20.65) | 20.18 (18.99- 21.37) |
| Other neurotic or anxiety disorder | 16.93 (16.86- 17.00) | 15.00 (14.73- 15.27) | 14.78 (14.50-15.06) | 16.61 (15.77- 17.45) |

^Notes: (1) Generalised anxiety disorder. (2) Obsessive-compulsive disorder. (3) Post-traumatic stress disorder.^

**Supplementary Table 6: Relative risk of a new diagnosis of anxiety at any time between age 18 and 27 years of age among those with ASD who had not been diagnosed with anxiety before the age of 18**

|  | **Cases versus population controls** | |
| --- | --- | --- |
|  | RR (95% CI)  Model 1 | RR (95% CI)  Model 2 |
| All ASD | 3.02 (2.82- 3.24) | 2.71 (2.52- 2.91) |
| ASD without ID | 3.54 (3.29- 3.82) | 3.13 (2.90-3.38) |
| ASD with ID | 1.84 (1.56- 2.18) | 1.70 (1.44- 2.02) |

^Notes: (1) Modified Poisson regression with cluster robust standard errors. Model 1 adjusted for age and sex. (2) Model 2 adjusted for age, sex, maternal and paternal age, parental educational attainment, family disposable income quintile, individual or parental migration, and maternal and paternal psychiatric history. (3) RR= Relative risk; CI = Confidence interval.^

**Supplementary Table 7: Proportion of the sample with and without an autism diagnosis who were diagnosed with anxiety before and/or after age 18**

| **Individuals with a diagnosis of autism** | | | |
| --- | --- | --- | --- |
|  |  | Diagnosed with anxiety after age 18 | |
|  |  | No % (n) | Yes % (n) |
| Diagnosed with anxiety before age 18 | No % (n) | 65.97 (2,671) | 15.58 (631) |
|  | Yes % (n) | 13.9 (563) | 4.54 (184) |

| **Individuals without a diagnosis of autism** | | | |
| --- | --- | --- | --- |
|  |  | Diagnosed with anxiety after age 18 | |
|  |  | No % (n) | Yes % (n) |
| Diagnosed with anxiety before age 18 | No % (n) | 87.44 (190,314) | 7.74 (16,852) |
|  | Yes % (n) | 3.84 (8,351) | 0.98 (2,128) |
